# CUL4B contributes to cancer stemness by repressing tumor suppressor miR34a in colorectal cancer

**DOI:** 10.1038/s41389-020-0206-3

**Published:** 2020-02-13

**Authors:** Yanjun Li, Huili Hu, Yuxing Wang, Yujia Fan, Yang Yang, Beibei Guo, Xueyong Xie, Jiabei Lian, Baichun Jiang, Bo Han, Yanlei Wang, Changshun Shao, Yaoqin Gong

**Affiliations:** 1 Key Laboratory of Experimental Teratology, Ministry of Education and Institute of Molecular Medicine and Genetics, School of Basic Medical Sciences, Shandong University, Jinan, Shandong China; 2 Key Laboratory of Experimental Teratology, Ministry of Education and Department of Pathology, School of Basic Medical Sciences, Shandong University, Jinan, Shandong China; 3 Department of Pathology, Qilu Hospital of Shandong University, Jinan, China; 4 Department of General Surgery, Qilu Hospital of Shandong University, Jinan, China; 5 State Key Laboratory of Radiation Medicine and Protection, Institutes for Translational Medicine, Soochow University, Suzhou, Jiangsu China

**Keywords:** Cancer stem cells, Non-coding RNAs

## Abstract

Given that colorectal cancer stem cells (CCSCs) play key roles in the tumor dormancy, metastasis, and relapse, targeting CCSCs is a promising strategy in cancer therapy. Here, we aimed to identify the new regulators of CCSCs and found that Cullin 4B (CUL4B), which possesses oncogenic properties in multiple solid tumors, drives the development and metastasis of colon cancer by sustaining cancer stem-like features. Elevated expression of CUL4B was confirmed in colon tumors and was associated with poor overall survival. Inhibition of CUL4B in cancer cell lines and patient-derived tumor organoids led to reduced sphere formation, proliferation and metastasis capacity. Mechanistically, CUL4B coordinates with PRC2 complex to repress miR34a expression, thus upregulates oncogenes including *MYCN* and *NOTCH1*, which are targeted by miR34a. Furthermore, we found that elevated CUL4B expression is associated with miR34a downregulation and upregulation of miR34a target genes in colon cancer specimens. Collectively, our findings demonstrate that CUL4B functions to repress miR34a in maintaining cancer stemness in CRC and provides a potential therapeutic target.

## Introduction

Colorectal cancer (CRC) is the main cause of cancer-related death, as a result of metastasis and chemoresistance^1,2^. Intratumoral heterogeneity that contributes to therapy failure was recently explained by CSCs model. CSCs refer to a subpopulation of immortal cells within the tumor that can self-renew and give rise to various differentiated cell types constituting the tumor. CSCs have high tumorigenic ability in generating xenograft tumors, which are highly invasive and metastatic, and are more resistant to chemotherapy and radiotherapy^3^. CSCs in colorectal cancer (CCSCs) can self-renew, undergo multilineage differentiation, and survive in adverse tissue microenvironments, and are responsible for chemoresistance and relapse^4^. Various molecules, including CD133, EphB2, ALDH, LGR5, and DCLK1 have been proposed as biomarkers for CCSCs^5,6^. Moreover, various pathways, such as WNT and NOTCH1 pathway, as well as the complex crosstalk between microenvironment and CSCs, were found to be involved in the fine-tuning of CCSCs compartment and regulate fate of CCSCs^7–9^.

The miR34a is a well-known tumor suppressor in various types of cancers^10^. Among its many functions, miR34a has been shown to limit self-renewal of cancer stem cells. In addition, miR34a also regulates stem cell differentiation and somatic cell reprogramming^11,12^. Many direct miR34a targets implicated in different biological pathways have been validated. For example, miR34a was shown to inhibit the cell cycle regulators cyclins, cyclin dependent kinases, and the proto-oncogene *MYCN*, which leads to proliferation inhibition^13^. miR34a also inhibits NOTCH signaling, which control cell-fate determination during development and oncogenesis^12^. miR34 expression is transactivated by p53, via p53 binding sites in its promoter^14^. Aberrant CpG methylation of its promoter also has been shown to silence miR34a in many types of cancers, including CRC^15,16^.

CUL4B acts as a scaffold protein that assembles DDB1, RBX1, and substrate receptors to form various CUL4B RING E3 ligase complexes (CRL4B)^17,18^. CRL4B catalyzes either polyubquitination for proteosomal degradation or monoubiquitination at H2A for epigenetic modification^19,20^. CUL4B is frequently overexpressed in multiple kinds of solid tumor and functions to promote cell cycle progression and metastasis by epigenetically repressing tumor suppressors including miRNAs^21–24^. Recent clinical study has suggested that miR34a contributes to CCSC proliferation and inhibit colon cancer stemness^11^. Strikingly, CUL4B is also a marker for predicting patient CRC outcome, but the mechanism has not been completely elucidated^25^. In this study, we tested whether *CUL4B* promotes CRC by supporting CRC stemness. We found that CUL4B complex directly targets the miR34a promoter for epigenetic silencing, and therefore represses transcription of miR34a that directly targets *MYCN*, *NOTCH1*, and *CD44*. These findings illuminate the role of *CUL4B* in CCSC maintenance and have therapeutic implications.

## Results

### Increased CUL4B expression is correlated with poor prognosis of CRC and promotes patient-derived organoid expansion

**Fig. 1:**
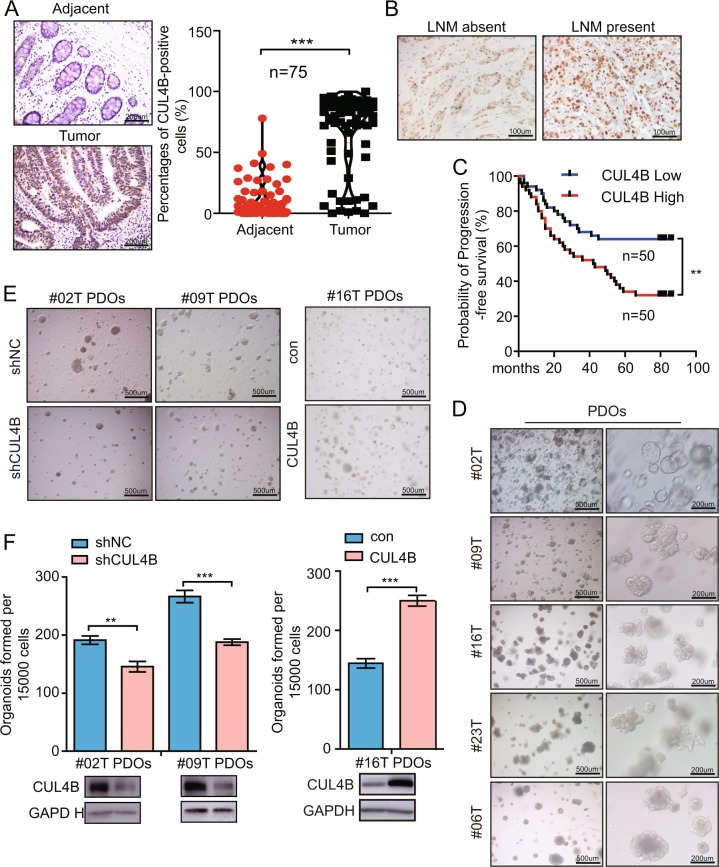
Increased CUL4B expression is correlated with poor prognosis of CRC and promotes patient-derived organoid expansion. **a** Representative pictures of IHC straining of CUL4B in human CRC tissues and the adjacent normal tissues (left). The percentages of CUL4B-positive cells in 75 paired human colon tumor and their adjacent tissues (right). Data represent mean ± SEM (*n* = 75). ***p < 0.001. **b** Representative pictures of IHC straining of CUL4B in human CRC LNM absent tissues and LNM present tissues. **c** Kaplan–Meier analysis of the correlations between CUL4B protein level and overall survival of 100 patients with CRC. CUL4B^high^, the expression of CUL4B is over than 50% in group and CUL4B^low^ is the other 50%. **p < 0.01. **d** Representative pictures of five PDOs. **e** Representative photographs were taken at ×40 magnification of organoids after the knockdown of *CUL4B* in #02T PDOs and #09T PDOs or after the overexpression of CUL4B in #16T PDOs. **f** Organoids formation assay showed organoid number per 15,000 cells in *CUL4B* knockdown and control #09T PDOs or #02T PDOs and in CUL4B overexpression and control #16T PDOs cultured for 7–10 days. Data represent mean ± SEM (*n* = 4). **p < 0.01; ***p < 0.001.

To address the role of CUL4B as a prognostic marker in CRC, we examined CUL4B expression by immunohistochemistry in tissue microarrays comprising tumor tissues and adjacent tissues from 75 cases of CRCs. As shown in Fig. 1a, CUL4B was significantly upregulated in 75 tumor tissues compared with paired adjacent tissues. Notably, primary tumors with lymph node metastasis (LNM) exhibited higher level of CUL4B expression than those without LNM (Fig. 1b and Supplementary Table S1). Furthermore, CUL4B expression levels were negatively correlated with survival status of CRC patients (Fig. 1c). Patient-derived tumor organoids (PDOs), which recapitulate many structural and functional aspects of tumors, are emerging models for cancer research and drug response prediction^26^. We then established five lines of CRC organoids (Fig. 1d) and evaluated the effect of CUL4B expression on tumor organoid-forming capacity. Knockdown of *CUL4B* in PDOs led to smaller tumor organoids and decreased organoid-forming capacity from single cells, whereas overexpression of CUL4B increased this capacity (Fig. 1e, f), suggesting that *CUL4B* plays oncogenic roles in CRC.

### *CUL4B* enhances CRC stemness

**Fig. 2:**
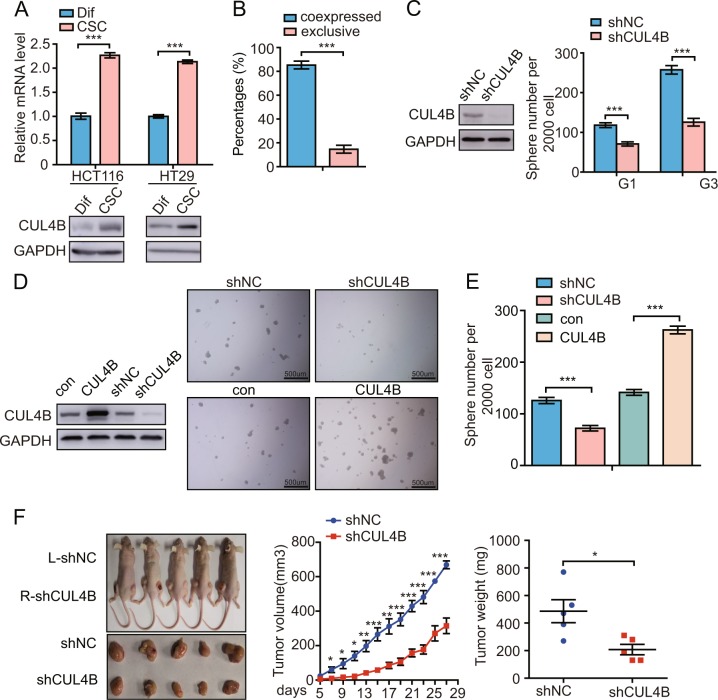
CUL4B enhances CRC stemness. **a** CUL4B expression level was determined in CCSCs and differentiated cancer cells, which were generated from CCSCs by culturing in 3% serum medium for 48 h, by western blot and qRT-PCR. ***p < 0.001. **b** Percentages of the HCT116 CSCs wherein ALDH1 and CUL4B were coexpressed or mutually exclusive by immunofluorescence staining. Coexpressed, ALDH1 co-stained with CUL4B; exclusive, ALDH1 was not co-stained with CUL4B. Data represent mean ± SEM (*n* = 3). ***p < 0.001. **c** Knockdown of *CUL4B* reduced the sphere formation ability of HCT116 cells. G1 generation 1, G3 generation 3. Sphere numbers per 2000 cells in *CUL4B* knockdown and control HCT116 cells cultured for 7 days. Data represent mean ± SEM (*n* = 6). ***p < 0.001. **d** Representative pictures of CCSCs after the knockdown or overexpression of CUL4B in HCT116 CSCs. Knockdown efficiency was confirmed by western blot at protein levels. **e** Effects of CUL4B knockdown or overexpression in HCT116 CSCs on sphere formation efficiency. Data represent mean ± SEM (*n* = 6). ***p < 0.001. **f** Knockdown of CUL4B in HT29-derived CSCs inhibited tumor growth. 1 × 10^6^ control cells (left leg) or *CUL4B* knockdown (right leg) HT29 CSCs were injected into nude mice (five mice per group), tumor growth was monitored from day 5 to day 25, and then tumors wee resected. (left) Representative pictures of nude mice and tumors from *CUL4B* knockdown or control HT29 CSCs (day 25). (middle) Growth curves of tumors formed by *CUL4B* knockdown (Red) or control HT29 CSCs (Blue) in nude mice. (right) Tumor weight of *CUL4B* knockdown (Red) or control HT29 CSCs (Blue) was measured on the day resected from mice. Data represent mean ± SEM (*n* = 5). *p < 0.05; **p < 0.01; ***p < 0.001.

The fact that *CUL4B* enhances the tumor-derived organoid-forming capacity suggested that *CUL4B* is involved in the enrichment of CSCs or cells with stem cell-related characteristics. To test this, we first examined whether CUL4B expression levels differ between CSCs and non-CSCs. CUL4B levels were significantly higher in the CSCs derived from HCT116 and HT29 cell lines than differentiated cells (Fig. 2a). Furthermore, the pair–cell assay indicated that CUL4B was highly coexpressed with ALDH1, a well-known CSCs marker, in CCSCs (Fig. 2b and Supplementary Fig. S1A, B). To further examine whether *CUL4B* regulates CCSCs, we first knocked down *CUL4B* in HCT116 cells. Serial sphere propagation assays showed that the knockdown of *CUL4B* strongly inhibited sphere formation capacity (Fig. 2c). Similar results were obtained with HT29 cells (Supplementary Fig. S1C). Consistently, knockdown of *CUL4B* in CCSCs derived from CRC cell lines reduced sphere numbers, whereas overexpression of CUL4B increased the ability of sphere formation (Fig. 2d, e and Supplementary Fig. S1D). Next, we used the mouse xenograft model to examine the effect of *CUL4B* knockdown on tumor growth by injecting *CUL4B* knockdown and control CCSCs into the left and right flank of the same nude mouse, respectively. As shown in Fig. 2f and Supplementary Fig. S1E, knockdown of *CUL4B* in HT29 and HCT116-derived CSCs led to smaller tumors than controls.

### *CUL4B* enhances metastatic capacity of patient-derived tumor organoids and CCSCs

**Fig. 3:**
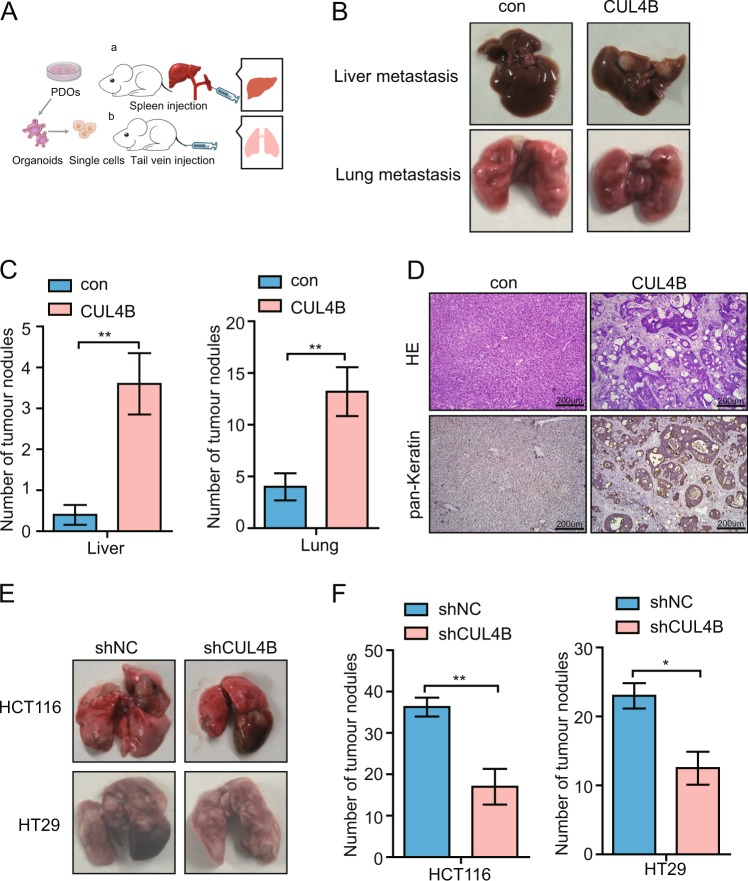
CUL4B enhances metastatic capacity of patient-derived tumor organoids and CCSCs. **a** (a) Schematic of the hepatic metastasis assay by splenic organoids injection, (b) Schematic of lung metastasis assay by tail vein injection. **b** Representative pictures of liver and lung metastasis of NCG mice with tumors from CUL4B overexpression and control #16T PDOs. **c** Numbers of tumor nodules in liver and lung metastasis of CUL4B overexpression #16T PDOs compared with control group. Data represent mean ± SEM (*n* = 5). **p < 0.01. **d** Representative pictures of HE and human pan-Keratin staining of liver metastasis of CUL4B overexpression and control #16T PDOs. **e** Representative pictures of lung metastasis of nude mice with *CUL4B* knockdown and control HCT116 or HT29 cells. **f** Numbers of tumor nodules in lung formed by *CUL4B* knockdown HCT116 or HT29 cells compared with control group. Data represent mean ± SEM (*n* = 4). *p < 0.05; **p < 0.01.

As cancer stem cells have been considered as seeds for tumor growth and metastasis, we further evaluated the effect of CUL4B expression on metastatic capacity of PDOs. To this end, CUL4B overexpression and control organoids were injected into spleen and tumor metastases to liver and lung were evaluated (Fig. 3a). We found that overexpression of CUL4B in organoids significantly increased metastatic potential to lung and liver in xenograft models, as demonstrated by increased number of micrometastatic nodules (Fig. 3b, c). Histological features of metastatic tumor organoids in engraftment liver were characterized by HE and human specific pan-keratin staining (Fig. 3d). We then tested lung metastasis of *CUL4B* knockdown and control colon cancer cell lines by tail vein injection. As shown in Fig. 3e, f, the number of tumor nodules was significantly reduced after the ablation of *CUL4B* in HCT116 and HT29 lines. Taken together, these data suggest that *CUL4B* promotes metastatic capacity and increases ability of tumor cells to colonize in the liver and lung.

### *CUL4B* positively regulates oncogene *MYCN* via repressing miR34a expression

**Fig. 4:**
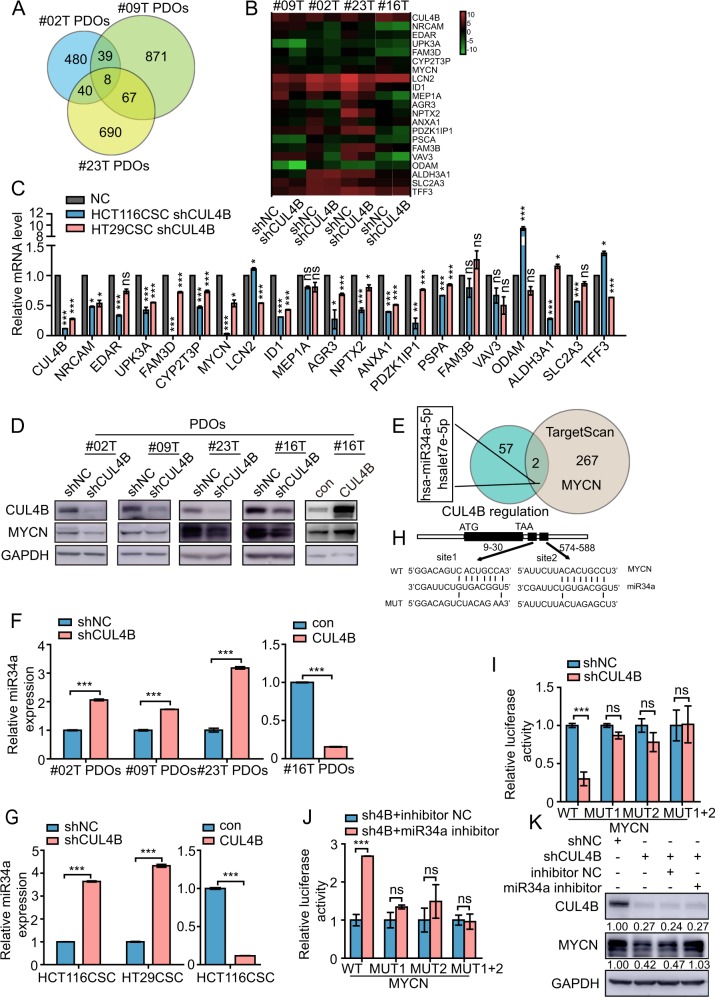
*CUL4B* positively regulates oncogene *MYCN* via repressing miR34a expression. **a** Venn diagram of downregulated genes by RNA sequencing in three CUL4B knockdown PDOs. **b** Heatmap of selected CUL4B-regulated candidate genes by RNA sequencing in CUL4B knockdown PDOs. **c** mRNA levels of CUL4B-regulated genes by qRT-PCR in CUL4B knockdown HCT116 CSCs and HT29 CSCs. Data represent mean ± SEM (*n* = 3). *p < 0.05; **p < 0.01; ***p < 0.001; ns no significance. **d** Western blotting analysis of MYCN expression in *CUL4B* knockdown or overexpressed PDOs. **e** Overlapping analysis of CUL4B-regulated candidate miRNAs with miRNAs targeting *MYCN*. **f** The levels of miR34a were analyzed by qRT-PCR in *CUL4B* knockdown or overexpressed and its control PDOs. Data represent mean ± SEM (*n* = 3). ***p < 0.001. **g** The levels of miR34a were analyzed by qRT-PCR in *CUL4B* knockdown or overexpressed and its control HCT116 CSCs and HT29 CSCs. Data represent mean ± SEM (*n* = 3). ***p < 0.001. **h** Schematic of two putative miR34a binding sites in the *MYCN* 3′UTR. **i** Luciferase assay showed reporter activity of wild-type *MYCN* 3′UTR, but not mutant, was decreased in CUL4B knockdown HCT116. Data represent mean ± SEM (*n* = 4). ***p < 0.001; ns no significance. **j** Luciferase assay showed decreased luciferase activity of wild-type, but not mutated *MYCN* 3′UTR in *CUL4B* knockdown HCT116 was rescued by miR34a repression. Data represent mean ± SEM (*n* = 4). ***p < 0.001; ns no significance. **k** Western blotting analysis shows decreased MYCN expression in *CUL4B* knockdown HCT116 cells was rescued by miR34a repression. Intensity of CUL4B and MYCN bands was qualified with GAPDH as the reference by Quantity One software. Reletavie number was listed below with shNC as the control.

In order to elucidate the regulation network of *CUL4B* in colon cancer regulation, we performed RNA sequencing of *CUL4B* knockdown and its control PDOs from three individuals. Eight genes were identified to be significantly downregulated by *CUL4B* knockdown in all three PDOs, including *NRCAM*, *EDAR*, *UPK3A*, *FAM3D*, *CYP2T3P*, *LCN2*, and *MYCN* (Fig. 4a). We also selected 13 well-known cancer-related genes that were obviously reduced in at least two of three *CUL4B* knockdown PDOs. Quantitative real-time PCR (qRT-PCR) was used to further examine the expression of these 20 genes in four *CUL4B* knockdown and their controls as well as *CUL4B* knockdown or control CCSCs. As shown in Fig. 4b, c, knockdown of *CUL4B* in PDOs or CCSCs significantly downregulated seven genes, including *ID1*, *UPK3A*, *CYP2T3P*, *FAM3D*, *NPTX2*, *AGR3*, and *MYCN*. Among these genes, *MYCN*, a well-recognized oncogene, is on the top of the downregulated genes in *CUL4B* knockdown PDOs and CCSCs, with significant downregulation in all five datasets. Western analysis further confirmed decreased MYCN protein level in all four *CUL4B* knockdown PDOs (Fig. 4d). Furthermore, MYCN was upregulated in CUL4B overexpressed PDOs (Fig. 4d).

It has been reported that several miRNAs target *MYCN*^13^, and our recent studies showed that CRL4B complex epigenetically represses miRNAs^23^. We thus performed miRNA sequencing in #16PDOs and identified 59 candidate miRNAs that might be regulated by CUL4B in colon cancer. Overlapping these miRNAs with the 269 miRNAs predicted to target *MYCN* by TargetScan database, miR34a and let7e were identified in both lists (Fig. 4e). qRT-PCR assay indicated that miR34a, but not let7e, was negatively regulated by *CUL4B* in both CCSCs and PDOs (Fig. 4f, g and Supplementary Fig. S2).

To further clarify whether *CUL4B* regulates MYCN expression through miR34a, we constructed luciferase reporter vectors containing 3′UTR of *MYCN* with and without point mutations in the two seed sequences of miR34a as previously described (Fig. 4h)^13^. Luciferase assay showed that the activity of the reporter containing the *MYCN* 3′UTR was much lower in *CUL4B* knockdown HCT116 than that in control cells (Fig. 4i). In contrast, no obvious difference in luciferase activity of mutant *MYCN* 3′UTR was observed between *CUL4B* knockdown and control cells. In addition, inhibition of miR34a could reverse the decreased activity of wild-type *MYCN* 3′UTR reporter (Fig. 4j). In line with these results, inhibition of miR34a was able to rescue the decreased levels of MYCN protein in *CUL4B* knockdown cells (Fig. 4k). Collectively, these results indicate that *CUL4B* positively regulates MYCN levels via repressing miR34a expression.

### CRL4B coordinates with PRC2 complex to repress miR34a expression

**Fig. 5:**
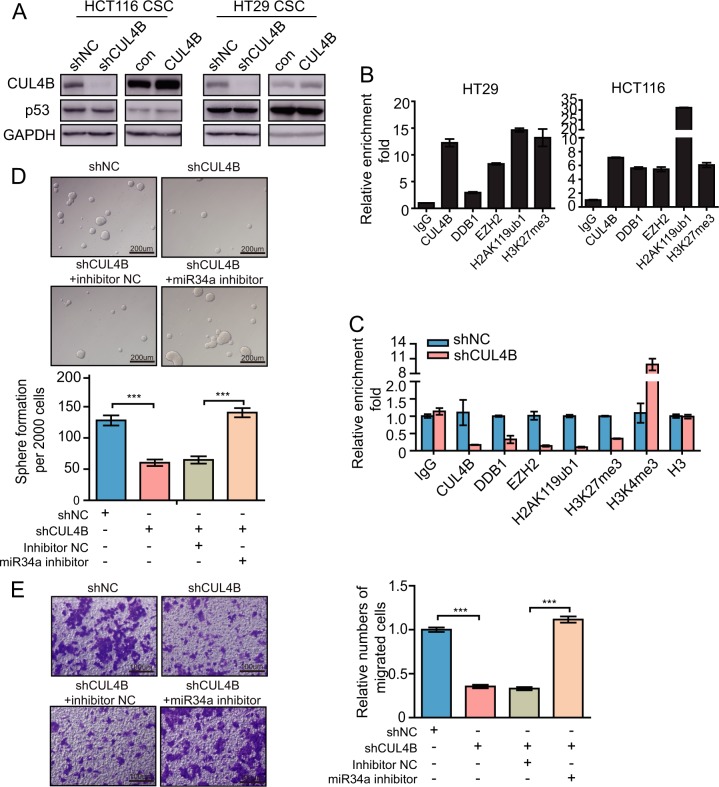
CRL4B coordinates with PRC2 complex to repress miR34a expression. **a** Western blotting analysis shows p53 protein level in *CUL4B* knockdown or overexpression CCSCs compared with its control cells. **b** ChIP assay was performed in HT29 and HCT116 with the antibodies of IgG, CUL4B, DDB1, EZH2, H2AK119ub, and H3K27me3. qRT-PCR was performed with primer at the region of miR34a promoter. **c** ChIP assay was performed in *CUL4B* knockdown and control HT29 with the antibodies of CUL4B, DDB1, EZH2, H2AK119ub, H3K27me3, H3K4me3, and Histone H3. qRT-PCR was performed with primer at the region of miR34a promoter. **d** Decreased sphere formation caused by *CUL4B* knockdown was rescued by miR34a repression in HT29 cells. Representative photographs were taken at ×100 magnification. Sphere formation numbers were counted in each treatment group after 7 days. Data represent mean ± SEM (*n* = 4). ***p < 0.001. **e** Decreased migration caused by *CUL4B* knockdown was rescued with miR34a inhibitor in HCT116 cells. Representative photographs were taken at ×200 magnification. Number of migrated cells were quantified in four random images from each treatment group cultured for 35 h. Data represent mean ± SEM (*n* = 4). ***p < 0.001.

miR34a has been reported as the target gene of p53^14,27^. To determine whether *CUL4B* regulates miR34a by regulating p53, we first evaluated the effect of manipulating CUL4B expression on p53 protein level in CCSCs. As shown in Fig. 5a, p53 levels in CCSCs were not altered by *CUL4B* knockdown or overexpression. Previous reports have shown that EZH2 binds to the promoter region of −1028 to −910 bp upstream of transcriptional starting sites of mature-miR34a^28,29^. We then performed ChIP assay with primer pairs covering that region. CUL4B, DDB1, and EZH2 co-occupancy was detected at this region and was associated with enriched H2AK119ub1 and H3K27me3, in both HCT116 and HT29 lines (Fig. 5b). Knockdown of *CUL4B* led to reduced binding of CRL4B and PRC2 complexes, consequently decreased H2AK119ub1, H3K27me3 and increased H3K4me3 (Fig. 5c and Supplementary Fig. S3A). Bisulfite sequencing revealed no alteration in the methylation pattern in the miR34a promoter after *CUL4B* knockdown (Supplementary Fig. S3B). Next, we examined the role of miR34a repression by *CUL4B* in maintaining the stemness of CRC cells. As shown in Fig. 5d, e, inhibition of miR34a remarkably restored the sphere-forming capacity and migration in *CUL4B* knockdown CRC cells, indicating that *CUL4B* contributes to maintaining CRC stemness at least partially through repression of miR34a.

### *CUL4B* upregulates miR34a targets in CCSCs, and its expression correlates with miR34a network in human colorectal cancer specimens

**Fig. 6:**
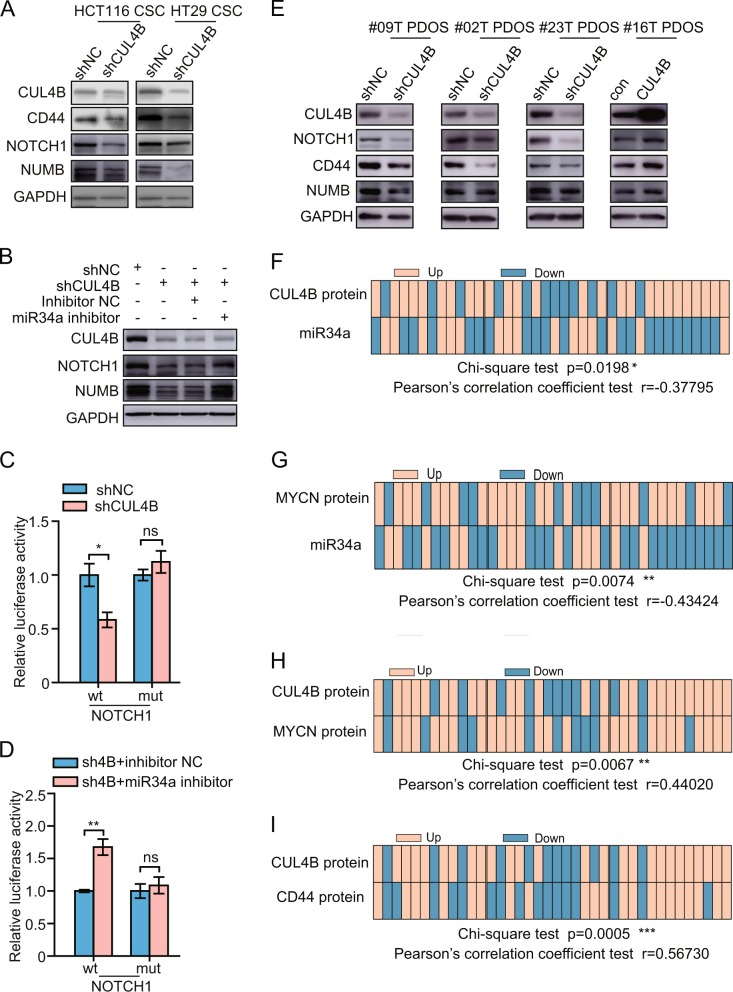
*CUL4B* upregulates miR34a targets in CCSCs, and its expression correlates with miR34a network in human colon cancer specimens. **a** Western blotting analysis to detect CD44, NOTCH1, and NUMB protein level in *CUL4B* knockdown and its control CCSCs. **b** Decreased NOTCH1 and NUMB expression in *CUL4B* knockdown HCT116 cells was rescued by miR34a repression. **c** Knockdown of CUL4B in HCT116 cells decreased wild-type, but not mutated, *NOTCH1* 3′UTR luciferase activity. Data represent mean ± SEM (*n* = 4). *p < 0.05; ns no significance. **d** Decreased luciferase activity of wild-type, but not mutated, *NOTCH1* 3′UTR was rescued by miR34a repression in *CUL4B* knockdown HCT116. Data represent mean ± SEM (*n* = 4). **p < 0.01; ns no significance. **e** Western blotting analysis shows CD44, NOTCH1, and Numb protein level in *CUL4B* knockdown or overexpressed PDOs. Distribution of CUL4B protein and miR34a (**f**), MYCN protein and miR34a (**g**), CUL4B protein and MYCN protein (**h**), CUL4B protein and MYCN protein (**i**) levels of 38 colorectal cancer samples; up or downregulation is relative to adjacent nontumor tissues.

The fact that *CUL4B* suppresses miR34a expression prompted us to examine whether other miR34a target genes are downregulated in *CUL4B* knockdown cells due to the derepression of miR34a. Indeed, the knockdown of *CUL4B* in CCSCs derived from HCT116 and HT29 cell lines significantly reduced the levels of NOTCH1, NUMB, and CD44 (Fig. 6a). Moreover, inhibition of miR34a could effectively restore these protein level in *CUL4B* knockdown HCT116 cells (Fig. 6b). The luciferase assay showed that the knockdown of *CUL4B* markedly suppressed the activities of reporter containing wild-type *NOTCH1* 3′UTR, whereas reporter carrying the mutant 3′UTR were unresponsive to *CUL4B* knockdown (Fig. 6c). Furthermore, inhibition of miR34a could reverse the decreased activity of wild-type *NOTCH1* 3′UTR-containing reporter (Fig. 6d). We also observed altered protein levels of CD44, NUMB, and NOTCH1 in *CUL4B* knockdown or overexpressed PDOs (Fig. 6e). Collectively these data supported the role of CUL4B in repressing miR34a, and thus upregulating genes that maintain CCSC features.

To further investigate the clinical relevance of our above findings, we isolated RNA and protein from 38 fresh tumor samples and paired adjacent normal tissues. As expected, downregulation of miR34a was observed in 61% (23/38) of tumor tissues (Supplementary Fig. S4A), while upregulation of CUL4B was observed in 68% (26/38) of cancer tissues (Supplementary Fig. S4B). Importantly, we observed a significant inverse correlation between CUL4B protein and miR34a levels (Fig. 6f). Moreover, upregulation of MYCN was observed in 71% (27/38) of cancer tissues (Supplementary Fig. S4C). Notably, miR34a was negatively correlated with MYCN (Fig. 6g), while positive correlation was detected between CUL4B and MYCN as well as CD44 (Fig. 6h, i). Collectively, these results support that CUL4B promotes tumorigenesis by regulating miR34 and its target genes.

## Discussion

CRC is highly lethal and has high rate of recurrence, in part because of CCSCs features^30,31^. CSCs appear to serve as critical drivers of tumor heterogeneity and malignancy in multiple solid tumors. In this study, we demonstrated that *CUL4B* acts to maintain CCSCs features through miR34a regulation. *CUL4B* promotes sphere formation from CRC cells and maintains CCSCs self-renewal. *CUL4B* promotes tumor expansion and metastasis capacity in xenograft. Mechanistically, CRL4B complex coordinates with PRC2 complex to repress miR34a expression thus upregulates miR34a target genes including *MYCN*. *CUL4B* upregulation is associated with miR34a downregulation and upregulation of miR34a targets in CRC tissues and poor survival rates, suggesting that elevated CUL4B expression can serve as predictive markers for patients with CRC.

miR34a is well-known tumor suppressor and tightly associated with cancer stem cell function^32^. miR34a expression leads to differentiation while its reduction to CCSC proliferation via symmetric self-renewal^11^. Loss of p53 function leads to downregulation of miR34a^14^. Also, miR34a tends to be silenced due to aberrant CpG methylation or histone modification by epigenetic mechanism by DNMT or PRC2 complex^15,16^. Although p53 could be downregulated by CUL4B in normal human fibroblasts under stress^33^, p53 protein level remained unchanged in CRC cells after *CUL4B* ablation. CpG methylation of miR34a promoter was not altered by knockdown of *CUL4B*. Instead, our results demonstrated an important role of CRL4B and PRC2 complexes in the transcriptional regulation of miR34a.

*MYCN*, a member of the MYC family of basic helix-loop-helix-zipper transcription factors, is well-established oncogene in several pediatric tumors^34,35^. *MYCN* has been shown to promote HCC proliferation and is positively associated with CSC markers^36^. *MYCN* downregulation broadly reverses tumor stem-like phenotypes and aberrant cell cycle in a variety of neuroblastoma models^37,38^. EZH2 is physically associated with MYCN with increased H3K27me3 at PRC2 target genes in pediatric cancers^35^. Besides, MYCN has also been reported to interact with EZH2 and its overexpression augments PRC2 signaling thus leading to the development of poorly differentiated and invasive prostate cancer^38^. Our results indicate that CRL4B complex coordinates with PRC2 complex to promote carcinogenesis^39^ and CCSCs features, it remains to be determined whether *MYCN* plays a role in recruiting CRL4B and thus forming a positive feedback loop.

In addition, CUL4B might promote CRC tumorigenesis by regulating other six genes we identified. Among them, NRCAM, the cell surface glycoprotein, has been reported to be significantly associated with nodal and distant metastasis in colon cancer. NRCAM is also an independent marker of poor prognosis among advanced CRC patients^40^. UPK3A, FAM3D, and LCN2, which participate cancer cell metabolisim, were also downregulated in all PDOs after CUL4B ablation. Further investigations are needed to understand whether CUL4B promotes tumorigenesis through other six genes by using more PDOs and specimens.

In conclusion, our study shows CRL4B complex coordinates with PRC2 complex to repress miR34a expression, thus upregulates the oncogenic miR34a targets, and consequently sustaining CCSCs features. This CUL4B-miR34a axis may have implications in CSC-targeting therapy of CRC.

## Materials and methods

### Tissue specimens

Three sets of tissue microarrays were used in this study. Two commercial tissue microarrays were purchased from Zuocheng (Shanghai, China). One microarray (CO150C01) containing 75 pairs of CRC and normal adjacent tissues was used to examine CUL4B expression. Three hundred cells from three randomly selected fields of each sample were analyzed for CUL4B-positive staining, and percentage of CUL4B-positive cells was calculated. The second microarray (CO180S10) included 100 CRC samples, 80 of which have paired adjacent tissues. The samples on C0180S10 microarray were collected in 2008. A follow-up survey of survival status was conducted in 2015. We used CO180S10 to examine the association of CUL4B expression with survival rates. The third microarray included 82 CRC patients who underwent surgery from 2013 to 2015 at Qilu Hospital of Shandong University (Jinan, China). None of the patients had received radiotherapy or chemotherapy prior to surgery. For RNA and protein analysis, a total of 38 pairs of human CRC and normal adjacent tissues were also obtained. This study was approved by School of Basic Medical Sciences Ethics Committee of Shandong University and informed consent was obtained from patients.

### IHC staining and analysis

IHC was performed according to the manufacture’s protocol as described before^41^. The stained sections were reviewed and scored independently by two pathologists from Qilu hospital of Shandong University.

### Generation of patient-derived organoids

Freshly collected tumor tissues were cultured into organoids as previously reported^42,43^. Briefly, isolated tissues were shortly incubated in 10 ml Ad-F+++ in Falcon tube. Advanced DMEM/F12 (Gibco) was supplemented with penicillin/streptomycin (Gibco), 10 mM HEPES (Gibco), and 2 mM GlutaMAX (Gibco). The tissue was cut into small pieces, washed with ice-cold type I solution at least five times, and subsequently digested with 500 mM EDTA for 25 min on the shaker. The supernatant was filtered, collected, and centrifuged. The cell pellet was suspended with matrigel (BD) and dispensed into two 24-well culture plates. The basal culture medium for organoids was prepared as following (50 ml): Ad-F+++ was supplemented with 1 ml B27 (Gibco), 500 μl N2 (Gibco), 10 nM gastrin I (RD), and 1.25 mM N-acetylcysteine (Sigma), 50 ng/ml recombinant EGF (Peprotech), 15% Noggin-conditioned medium, 30% R-spondin-1-conditioned medium, 50% Wnt3A-conditioned medium, 500 nM A83-01 (Sigma), 3 mM SB202190 (Sigma), and 10 nM Y-27632 2HCl (Selleck).

### Tumor organoid-forming assay

The tumor organoids were collected from matrigel by incubation with Cell Recovery Solution (Corning). Organoids were digested with Trypsin-EDTA (0.25%). Single cells were counted and resuspended in 500 μl of culture medium with 4–8 MOI of CUL4B knockdown or control virus, and 8 μg/mL Polybrene. Cells were transferred into one well of a 24-well plate and were incubated at 37 °C 5% CO_2_ for 24 h. Cells were collected and resuspended in Matrigel and were replated in 60 μL droplets in each well of 24-well plates. A total of 2 μg/ml puro (Invivogen) were added to culture medium on day 5 for selection. After 3–5 weeks culture, the same number of single cells (15,000) from CUL4B knockdown/overexpression or control organoids were resuspended in 60 μl of Matrigel and replated per well. After 7–14 days, the number of re-formed tumor organoid (≥50 μm) was counted and analyzed.

### Cell culture and manipulation

The HCT116 and HT29 CRC cell lines were purchased from the Shanghai Cell Collection (Chinese Academy of Sciences). All cell lines were cultured according to the manufacturer’s specifications in RPMI 1640 medium (Gibco) supplemented with 10% FBS (AusGeneX). Cells were maintained at 37 °C in a humidified atmosphere with 5% CO_2_. HCT116 and HT29 cell lines were tested for STR and mycoplasma.

*CUL4B* knockdown (shCUL4B) and its control cells (shNC), or CUL4B overexpression (CUL4B), and its control cells (con) were generated as described previously^22^, the constructs are shown in Supplementary Fig. S5. We plated shNC or purified shCUL4B CRC cells 24 h before transfection. Cells were transfected with 20 nM of miR34a inhibitor or non-targeting inhibitor negative control miRNA oligos purchased from RiboBio (Guangzhou, China) by using lipofectamine 2000 (Invitrogen). Alternatively, we transfected con or CUL4B CRC cells with 20 nM of miR34a mimics or mimics negative control miR-NC purchased from RiboBio (Guangzhou, China). The cells were harvested 48 h post transfection.

### CCSC enrichment, culture and sphere formation

CCSC sphere formation, enrichment and culture were performed as described previously^11,44,45^. We generated spheroid-derived CCSCs from HCT116 and HT29 CRC cells. Briefly, cells were dissociated using 0.25% trypsin-EDTA. 5 × 10^6^ single cells were plated on 10 cm Costar ultralow attachment flasks (Corning) in DMEM/F12 stem cell medium containing N2 supplement (Invitrogen), B27 supplement (Invitrogen), EGF (40 ng/ml), and bFGF (20 ng/ml). Cells were cultured at 37 °C at 5% CO2 for 5–7 days to form spheres. Spheres were then collected at 500 rpm for 3 min and dissociated using 0.25% trypsin-EDTA. Single cells were passaged at a ratio of 1:3. CCSCs were further enriched by passaging 7–10 generations. All CCSCs we used in our experiments were over eight passages.

### Spheroid formation assays

Single cells of *CUL4B* wild-type or knockdown HCT116 and HT29 were counted and cultured in serum-free stem cell medium in Costar ultralow attachment flasks (Corning) for 5–7 days. Spheres with a diameter over 50 μm were counted as generation one (G1). Then these spheres were digested into single cells and passaged. After two passages, the same number of single cells from *CUL4B* wild-type and knockdown HCT116 and HT29 spheres were seeded and allowed to re-form spheres. Re-formed spheres with a diameter over 50 μm were counted as generation three (G3).

### Tumor xenografts

BALB/c nude mice (male) were purchased from the Model Animal Resource Information Platform (Nanjing, China). The mice were randomly divided into two groups. HCT116 CSCs or HT29 CSCs stably transfected with CUL4B or control shRNA were collected. A total of 1 × 10^6^ viable cells in 200 ul PBS were injected subcutaneously into 6-week-old BALB/c male nude mice. Visible tumors were measured every 2 days using a vernier caliper, and the volume was calculated according to the formula: 1/2 length × square width. All animal experiments were carried out upon approval of the Animal Care and Use Committee of the School of Basic Medical Sciences, Shandong University.

### Organoid xenotransplantation

Organoid xenotransplantation assay was performed as described previously^42,46^. NOD-Prkdcem26Il2rgem26/Nju mice (NCG, male, 6 weeks old) were purchased from the Model Animal Resource Information Platform (Nanjing, China). The mice were randomly divided into two groups. The expanded organoids were harvested. A total 25 ul (1 × 10^6^ cells) of Matrigel-organoid suspension were injected into the spleens of NCG mice. The mice were euthanized 3 months after transplantation. The livers and lungs were collected, and tumor numbers were calculated. Each PDO was transplanted into five independent NCG mice. Tissue samples were then formalin-fixed and embedded in paraffin for subsequent immunohistochemical analysis.

### Cell migration assay

Transwell inserts for 24-well plates with porous filters without coating (the pore size was 8 μm) were used for the evaluation of cell migration. A total of 1.5 × 10^5^ cells in 200 μl serum-free RPMI 1640 (Gibco) were seeded into the inserts. And then 600 μl RPMI 1640 (Gibco) with 10% FBS was added in the lower portion of the chamber as a chemoattractant. After 35 h of incubation the cells that transferred to the lower well of the chamber were stained using crystal violet.

### Western blot

Total proteins were extracted from the cells using RIPA buffer (Keygen) with 1 mM proteinase inhibitor PMSF (Keygen) and cocktail (Roche). Forty micrograms of protein were separated on a polyacrylamide gel and transferred to a nitrocellulose membrane. The membranes were blocked for 1 h at room temperature in TBST containing 5% BSA, and then incubated overnight at 4 °C in TBST containing 5% BSA and following antibodies: CUL4B (Sigma, C9995); CD44 (Sigma, HPA005785 or Abcam, ab157107); NOTCH1 (CST, 3608S); NUMB (Abcam, ab4147); MYCN (Genetex, GTX133721); and GAPDH (CST, 5174S). Membranes were washed in TBST, incubated with a secondary antibody and conjugated with horseradish peroxidase for 1 h at room temperature. After washes with TBST, bands were detected using a SuperSignal Chemiluminescence kit (Thermo). Intensity of bands was qualified with GAPDH as the reference by Quantity One software.

### RNA extraction and quantitative real-time PCR

Total RNA extraction, reverse transcription PCR, and qRT-PCR assay were performed as described previously^21^. Total RNA was extracted with Trizol reagents (Thermo) following the manufacturer’s instructions. The mRNA levels of genes and mature miRNA were assayed by SYBR Green PCR kit (Roche). Actin and U6 were used as the endogenous control for mRNA and miRNA, respectively. The primer sequences were listed in Supplementary Table S2. Primers used for miRNA qRT-PCR were purchased from GenePharma (Shanghai, China).

### Pair–cell assay

Pair–cell assay was performed as described previously^11^. Briefly, CCSCs (enrichment over ten passages from tumor cells) were dissociated from spheres using trypsin-EDTA. Single cells were plated at 1.8 × 10^5^ cells/mL on 60 mm glass culture slide (pre-coated with poly-lysine or BD, Falcon 354114) and allowed to progress through one cell division for 24 h. After being fixed in 4% paraformaldehyde, the cells were blocked in 10% normal goat serum for 1 h at room temperature. The cells were incubated with antibodies against ALDH1 (Santa, sc-374149), and CUL4B (Sigma, C9995) overnight at 4 °C. Then, the cells were incubated with labeled secondary antibody (Invitrogen) for 1 h at room temperature. After being counterstained with DAPI (Sigma), the slide was observed under a fluorescent microscope (Olympus). Cells were analyzed for CUL4B and ALDH1 costaining (coexpressed) or single staining (exclusive).

### RNA sequencing

Total RNA of PDOs was extracted with Trizol reagents (Thermo) following the manufacturer’s instructions. A total of eight RNA samples were processed using an Illumina Hiseq 2500 platform (Novogene, Beijing, China). RNA-seq generated ~6.0 Gb of sequencing data with 150-bp paired-end reads for each sample. miRNA-seq generated ~0.5 Gb of sequencing data with 50-bp single reads for each sample. All RNA-seq and miRNA-seq data supporting this article are accessible through NCBI’s gene Expression Omnibus accession number GSE143505.

### Luciferase assays

*MYCN* 3′UTR or *NOTCH1* 3′UTR were cloned into the pmir-GLO vector. The pmir-GLO-MYCN 3′UTR vector or pmir-GLO-NOTCH1 3′UTR vector containing mutated miR34a binding site was generated by site-directed mutagenesis using overlap extension PCR. The reporter luciferase assays were performed as previously described^19^. The primer sequences were listed in Supplementary Table S3.

### Chromatin immunoprecipitations (ChIPs)

ChIPs were performed as described previously^19^. The Antibodies are CUL4B (Sigma, C9995), DDB1 (Santa, sc-137132), H3K27me3 (CST, 9733S), H3K4me3 (CST, 9751S), EZH2 (CST, 5246S), H2AK119ub1 (CST, 8240S), and H3 (Abcam, ab1791). The primer sequences are listed in Supplementary Table S4.

### DNA isolation and methylation analyses

Genomic DNA from CCSCs was isolated using the DNeasy Blood and Tissue Kit (Qiagen). Bisulfite modification was performed with the EpiTect Bisulfite Kits (Qiagen) according to the manufacturer’s instructions. We detected methylation of miR34a CpG island (GGCGCGCCCCGCGACCCAGCGGCGGCGTGGGCGAGGGGCGCTGCG). Methylation analyses were done by Gene Tech (Shanghai, China). The primer sequences are listed in Supplementary Table S4.

### Statistical analysis

Statistical analysis was performed using unpaired Student’s *t* test to calculate a two-tailed *P* value between two groups. Differences were considered significant at *P* < 0.05. The data are recorded as the mean ± SEM.

## Supplementary information

Supplementary Table S1-S4

Supplementary Figure Legends

Supplementary Figure 1

Supplementary Figure 2

Supplementary Figure 3

Supplementary Figure 4

Supplementary Figure 5
