# Supplementary material for: CUL4B contributes to cancer stemness by repressing tumor suppressor miR34a in colorectal cancer
Source: Oncogenesis. 2020 Feb 13;9(2):20. doi: 10.1038/s41389-020-0206-3 (PMC7018700; doi:10.1038/s41389-020-0206-3)
Supplement: Supplementary file 1 — Supplementary Table S1-S4 [file 41389_2020_206_MOESM1_ESM.doc]

**Supplemental materials**

Table S1 Clinicopathological analysis of CUL4B expression in CRC

| Variables | **CUL4B expression (%)** | | ***P*** |
| --- | --- | --- | --- |
|  | **Negative and weak** | **Moderate and strong** |  |
| **Age(years)** |  |  |  |
| <65 | 31 (58.5) | 22 (41.5) | 0.349 |
| ≥65 | 20 (68.9) | 9 (30.1) |  |
| **Sex** |  |  |  |
| Male | 28 (59.6) | 19 (40.4) | 0.571 |
| Female | 23 (65.7) | 12 (34.3) |  |
| **Tumour size(cm)** |  |  |  |
| <3 | 21 (72.4) | 8 (27.6) | 0.158 |
| ≥3 | 30 (56.6) | 23 (43.4) |  |
| **Differentiation** |  |  |  |
| Well to Moderate | 25 (71.4) | 10 (28.6) | 0.136 |
| Poor | 26 (55.3) | 21 (44.7) |  |
| **Tumor invasion depth** |  |  |  |
| T1-T2 | 24 (72.7) | 9 (27.3) | 0.106 |
| T3-T4 | 27 (55.1) | 22 (44.9) |  |
| **Lymph node metastasis (LNM)** |  |  |  |
| Absent(M0) | 34 (72.3) | 13 (27.7) | **0.028** |
| Present(M1) | 17 (48.6) | 18 (51.4) | ***P<0.05** |

Table S2 The primer sequences for qRT-PCR .

| CUL4B F | TGGAAGTTCATTTACCACCAGAGATG |
| --- | --- |
| CUL4B R | TTCTGCTTTTAACACACAGTGTCCTA |
| Actin F | TGACGGGGTCACCCACACTGTGCCCATCTA |
| Actin R | CTAGAAGCATTTGCGGTGGACGATGGAGGG |
| LCN2 F | ACTCGCCACCTCCTCTTCCACC |
| LCN2 R | GGCCTACCACATACCACTTCCCC |
| ID1 F | GCTGCTCTACGACATGAACGGCT |
| ID1 R | GCTTCAGCGACACAAGATGCGAT |
| MEP1A F | TCTGTATGCCTTTGAGATGTTCCGT |
| MEP1A R | CCGTCCTTGACTGCTCGTGGT |
| AGR3 F | TCTTACTCGTCACAGTTTCTTCCAA |
| AGR3 R | ATTCTGAGCCATTTCTTGTATTTCTT |
| NPTX2 F | GCAAGGTGGCAGAGCTGGAG |
| NPTX2 R | ACAGGCAGATGGTGAAGGCGTA |
| EDAR F | AGCAAGGACGAGGAGAAGAAAGAG |
| EDAR R | AACCAGCGACAGCAGGCACAG |
| FAM3D F | CTGGACAGGATGAGAGTGTCAGGT |
| FAM3D R | GCAGATTTTAAACGCAAAGTAGTTGG |
| CYP2T3P F | CACTGGAAGGTATTTGGGTGGAA |
| CYP2T3P R | GAACTCATGATGCGGAAGTTGTC |
| NRCAM F | AGCCATCCACCATACCATTTCTG |
| NRCAM R | TCTATTTTTCTGCTGGGGTCATCA |
| MYCN F | TGTCACCACATTCACCATCACTG |
| MYCN R | ACGCCTCGCTCTTTATCTTCTTC |
| UPK3A F | CAACAACCCCACACTTACCACT |
| UPK3A R | ATCAGAACTCCCCATGTCCAC |
| ANXA1 F | TGCTTTCTCTTGCTAAGGGTGAC |
| ANXA1 R | CTTCTGGTGGTAAGGATGGTATTG |
| PDZK1IP1 F | TCCTGGTCCTCGTTGCAATCG |
| PDZK1IP1 R | GCCTTCCTCCTCGGGCACATT |
| PSCA F | CAAAGCCCAGGTGAGCAACGA |
| PSCA R | GAGTCATCCACGCAGTTCAAGC |
| FAM3B F | AGGTGGTGTTCGTGGTCTTCGC |
| FAM3B R | GTCACATTTTTGCCTTTTGGGG |
| VAV3 F | TACTACAAGCATCATTCTCTCAAGGA |
| VAV3 R | CAACAAGGACAACTCTCTCATATCTC |
| ODAM F | ATTCCAGGACTCTCCCAGTTCTCTT |
| ODAM R | GTTGTGTCTGAGGCGGTGTTTGA |
| ALDH3A1 F | CTCCAGCAACGACAAGGTGATTAA |
| ALDH3A1 R | AAGAGCGGCGGTGAGAGAAAGT |
| SLC2A3 F | TGGGTTTTACCATCCTTCCTGCT |
| SLC2A3 R | TTGCTTTTCTTGTGACATCCTTGC |
| TFF3 F | TCCCGGAGCCCACGGTGGTCAT |
| TFF3 R | TTGGCTGGCACGGCACACTGGT |
| miR34a-5p F | TCTGTCTCTCTTGGCAGTGTCTTA |
| miR34a-5p R | AATGGTTGTTCTCCACTCTCTCTCTC |
| let7e-5p F | CATTCTCTCAGATGAGGTAGGAGG |
| let7e-5p R | TATGGTTTTGACGACTGTGTGAT |
| U6 F | CAGCACATATACTAAAATTGGAACG |
| U6 R | ACGAATTTGCGTGTCATCC |

Table S3 The primer sequences for construction of luciferase reporters

| NOTCH1 wt F | CGAGCTCCTCGCAGCACAGCTACT |
| --- | --- |
| NOTCH1 wt R | GCTCTAGATTCAACTTCCCTTCTCCAACAT |
| NOTCH1 mut F | ACTTTTATTTTACACAGAAAGAGTCGCTTTTTA |
| NOTCH1 mut R | TAAAAAGCGACTCTTTCTGTGTAAAATAAAAGT |
| MYCN wt F | CGAGCTCACGCTTCTCAAAACTGGACAGTCA |
| MYCN wt R | CCTCGAGTATGTATCAGCGTCATACTAAAGTAT |
| site1 MYCN mut F | ACTGGACAGTCTACAGAACTTTGCACATTT |
| site1 MYCN mut R | AAATGTGCAAAGTTCTGTAGACTGTCCAGT |
| site2 MYCN mut F | CTAATTCTTACTAGAGCTGTATACTTTAGTATG |
| site2 MYCN mut R | CATACTAAAGTATACAGCTCTAGTAAGAATTAG |

Table S4 The primer sequences for ChIP assay and methylation analyses

| miR34a CHIP F | CCGTGCGCGCAGGGCAGCGTGGAAA |
| --- | --- |
| miR34a CHIP R | CCAGAGACGCAAAGTCGGACGCGGGT |
| F-miR34a | GGAGAGGAGTTTTAGGAAGGGT |
| R-miR34a | AACACCAAATCCTAATCCTCAT |
| S-miR34a | AGTTAGGGATTTYGGTTTTGGGTA |
