# Supplementary material for: CUL4B contributes to cancer stemness by repressing tumor suppressor miR34a in colorectal cancer
Source: Oncogenesis. 2020 Feb 13;9(2):20. doi: 10.1038/s41389-020-0206-3 (PMC7018700; doi:10.1038/s41389-020-0206-3)
Supplement: Supplementary file 2 — Supplementary Figure Legends [file 41389_2020_206_MOESM2_ESM.doc]

**Supplementary Figure 1.**

**A,** Representative pictures of CCSCs costained with antibodies against ALDH1 and CUL4B. **B,** Percentages of HT29 CSCs wherein ALDH1 and CUL4B were coexpressed or mutually exclusive by immunoﬂuorescence staining. Data represent mean ± SEM (n=3). ***, p<0.001. **C,** Knockdown of *CUL4B* reduced sphere formation ability of HT29 cells into CCSCs under ultralow attachment culture. G1, generation 1, G3 generation 3. sphere number per 2000 cells in *CUL4B* knockdown and control HT29 cells after 7 days. Data represent mean ± SEM (n=6). ***, p<0.001. **D,** Representative pictures of CCSCs after knockdown of *CUL4B* in HT29 CSCs. Knockdown efficiency was confirmed by Western blot at protein levels. Sphere formation numbers of HT29 CSCs were counted in *CUL4B* knockdown compared with control cells after 5 days. Data represent mean ± SEM (n=6). ***, p<0.001. **E,** Knockdown of CUL4B in HCT116-derived CSCs inhibited tumor growth. 1000k control cells (left leg) or *CUL4B* knockdown (right leg) HCT116 CSCs were injected into nude mice , tumor growth was monitored from day 14 to day 34, and tumors were resected. **left,** Representative pictures of nude mice and tumors from *CUL4B* knockdown or control HCT116 CSCs (Day 34). **middle,** Growth curves of tumors formed by *CUL4B* knockdown (Red) or control HCT116 CSCs (Blue) in nude mice. **right,** Tumor weight of *CUL4B* knockdown (Red) or control HCT116 CSCs (Blue) was measured on the day resected from mice. Data represent mean ± SEM (n=5). *, p<0.05; **, p<0.01; ***, p<0.001.

**Supplementary Figure 2.**

The levels of let7e were analyzed by qRT-PCR in *CUL4B* knockdown or overexpression and its control PDOs. Data represent mean ± SEM (n=3). **, p<0.01; ***, p<0.001.

**Supplementary Figure 3.**

**A,** ChIP assay was performed in *CUL4B* knockdown and control HCT116 with the antibodies of CUL4B, DDB1, EZH2, H2AK119ub, H3K27me3 and H3K4me3. **B,** Sequencing map of DNA methylation after bisulfite transformation in *CUL4B* knockdown and control HCT116 CSCs and HT29 CSCs.

**Supplementary Figure 4.**

**A,** miR34a levels in 38 paired colorectal cancer samples by qRT-PCR. Data represent mean ± SEM (n=3). *, p<0.05; **, p<0.01; ***, p<0.001; ns, no significance. **B,** CUL4B, CD44, NOTCH1 and NUMB protein levels in 38 paired colorectal cancer samples by Western blot. **C,** MYCN protein levels in 38 paired colorectal cancer samples by Western blot.

**Supplementary Figure 5.**

The schematic of lentiviral constructs used for CUL4B knockdown and overexpression.
