# Supplementary figures and images for: CUL4B contributes to cancer stemness by repressing tumor suppressor miR34a in colorectal cancer
Source: Oncogenesis. 2020 Feb 13;9(2):20. doi: 10.1038/s41389-020-0206-3 (PMC7018700; doi:10.1038/s41389-020-0206-3)

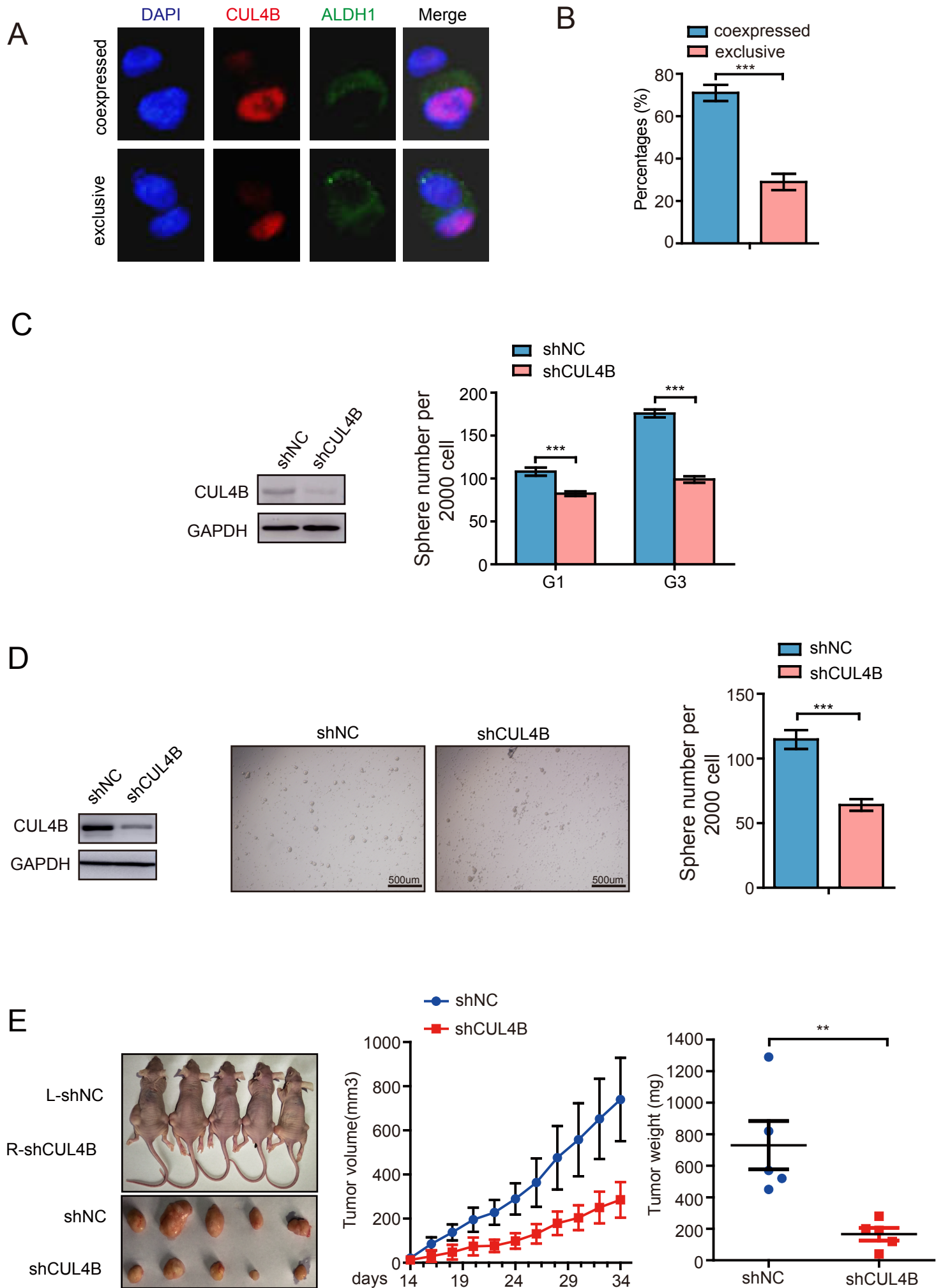

Supplement: Supplementary file 3 — Supplementary Figure 1 [file 41389_2020_206_MOESM3_ESM.pdf]

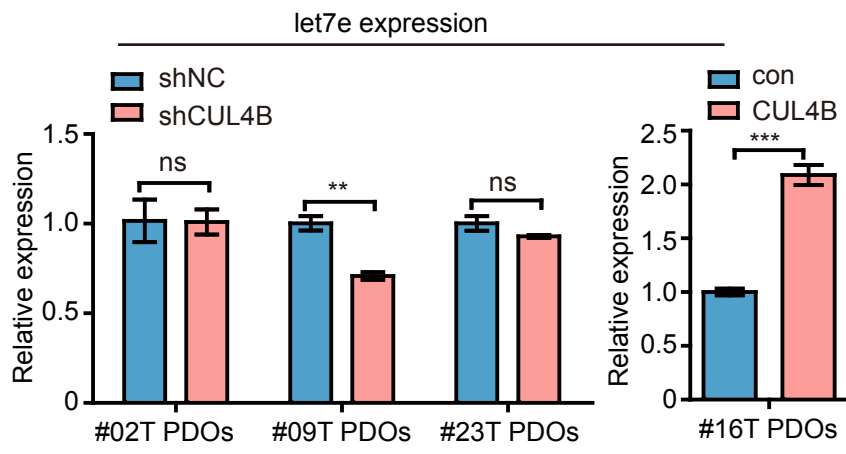

Supplement: Supplementary file 4 — Supplementary Figure 2 [file 41389_2020_206_MOESM4_ESM.pdf]

A

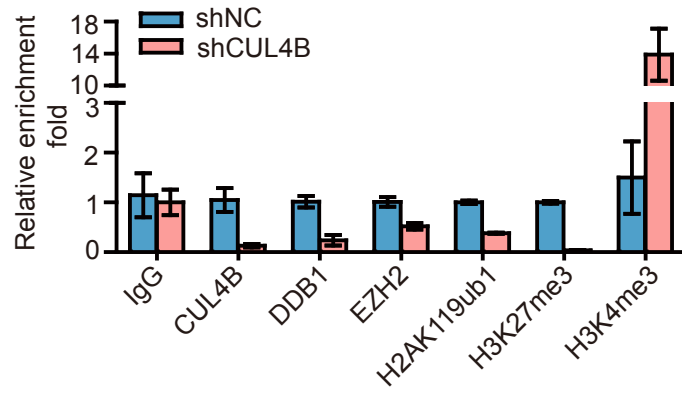

B

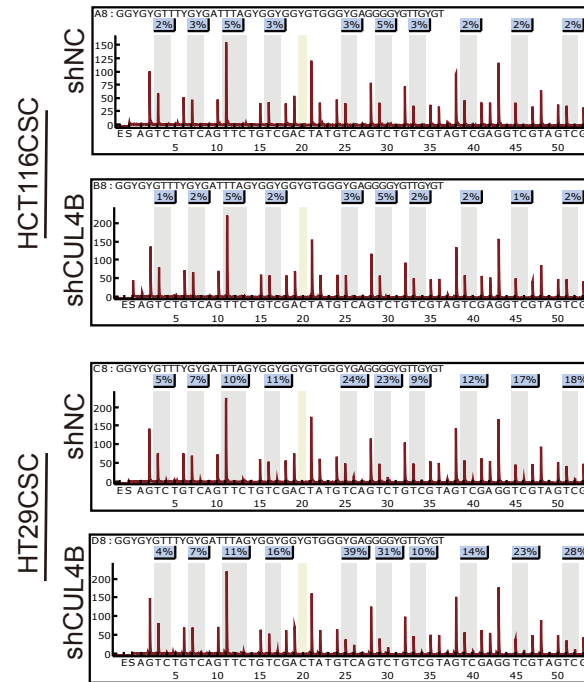

Supplement: Supplementary file 5 — Supplementary Figure 3 [file 41389_2020_206_MOESM5_ESM.pdf]

**A**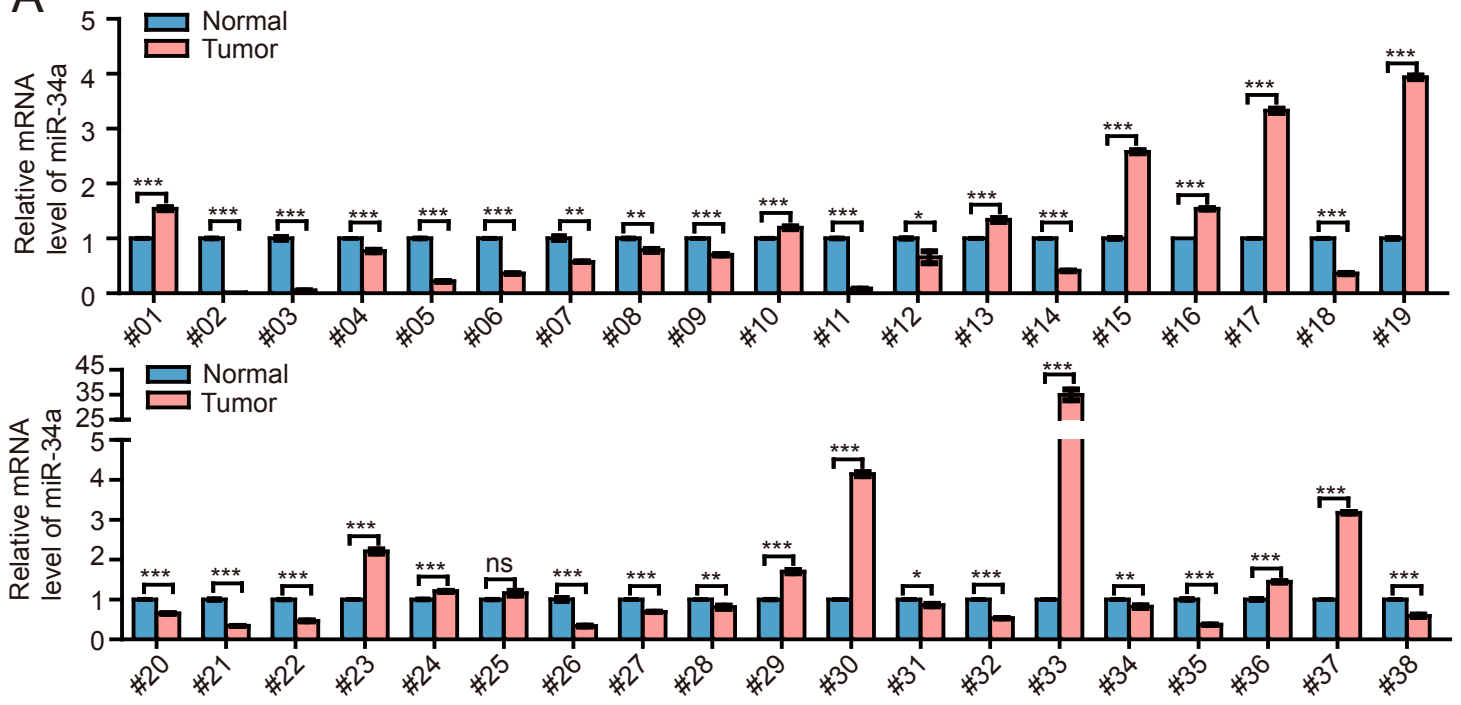**B**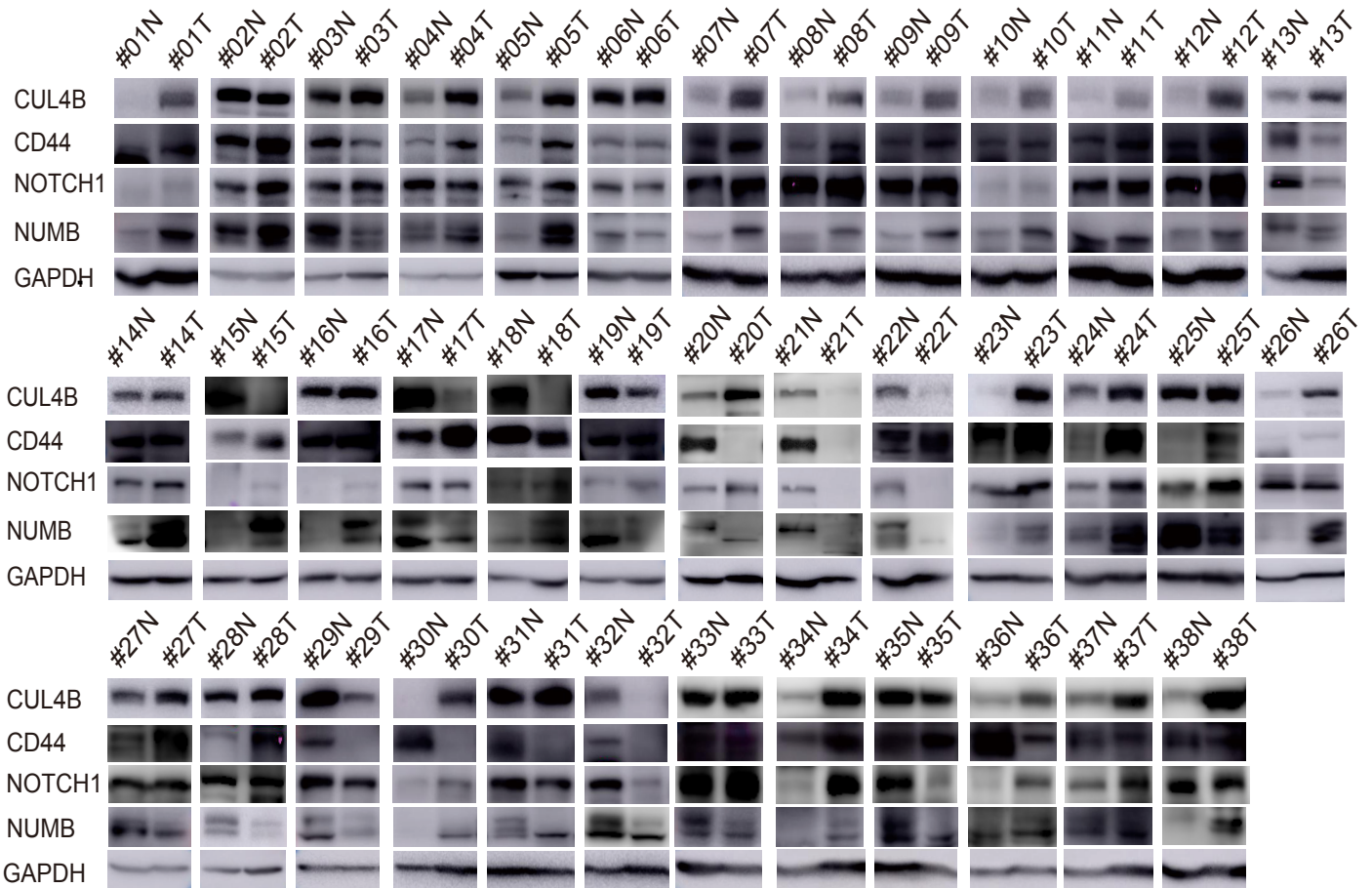**C**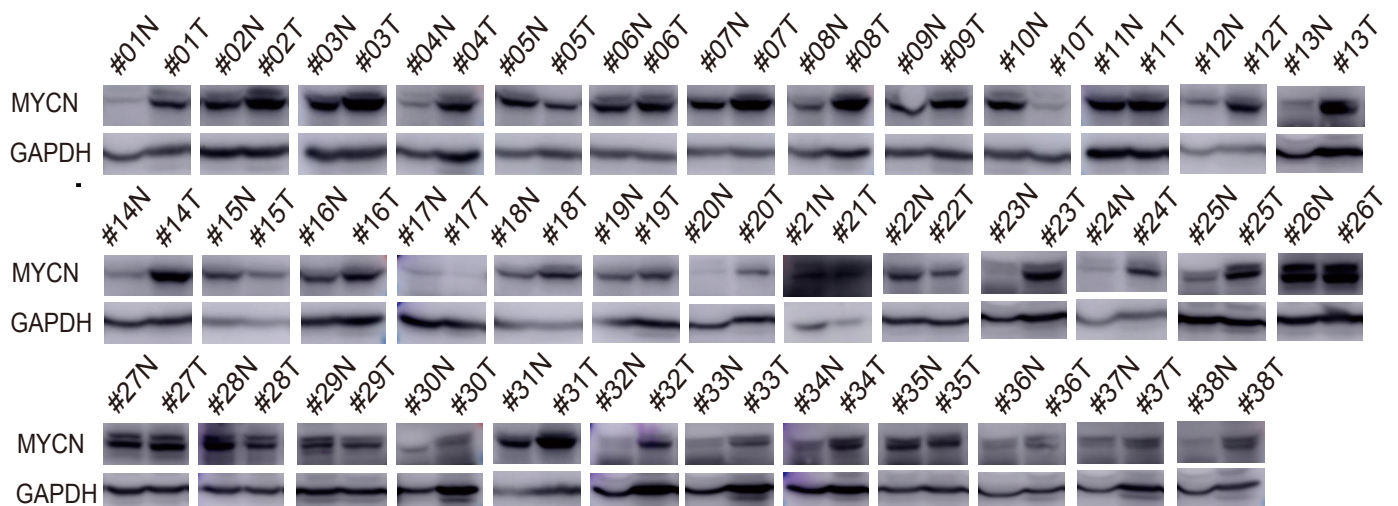

Supplement: Supplementary file 6 — Supplementary Figure 4 [file 41389_2020_206_MOESM6_ESM.pdf]

Supplementary Figure 5

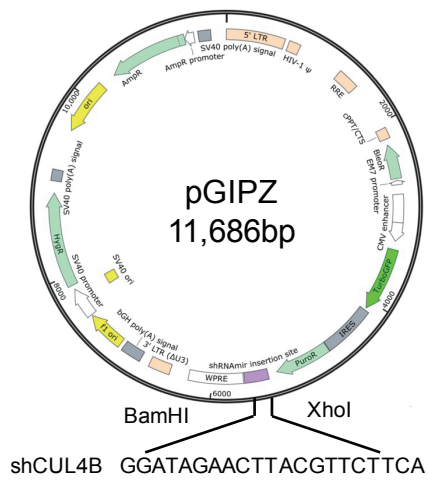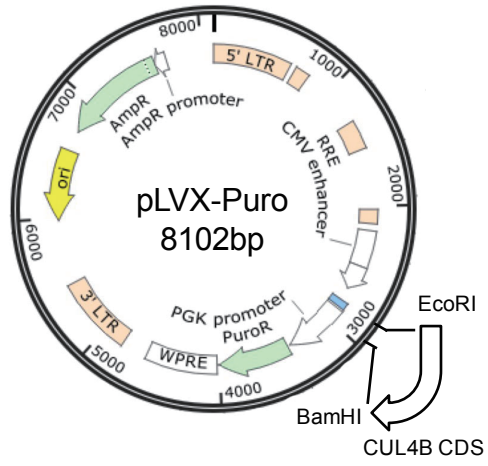

Supplement: Supplementary file 7 — Supplementary Figure 5 [file 41389_2020_206_MOESM7_ESM.pdf]
